# Primary healthcare policy implementation in the Eastern Mediterranean region: Experiences of six countries

**DOI:** 10.1080/13814788.2017.1397624

**Published:** 2017-11-23

**Authors:** Chris van Weel, Faisal Alnasir, Taghreed Farahat, Jinan Usta, Mona Osman, Mariam Abdulmalik, Nagwa Nashat, Wadeia Mohamed Alsharief, Salwa Sanousi, Hassan Saleh, Mohammed Tarawneh, Felicity Goodyear-Smith, Amanda Howe, Ryuki Kassai

**Affiliations:** ^a^ Department of Primary and Community Care, Radboud University Medical Centre Nijmegen The Netherlands; ^b^ Department of Health Services Research and Policy, Australian National University Canberra Australia; ^c^ Department of Primary Care and Public Health, Imperial College London UK; ^d^ Department of Family Medicine, Menoufia University Menoufia Egypt; ^e^ Department of Family Medicine, American University of Beirut Beirut Lebanon; ^f^ Primary Health Care Corporation Doha Qatar; ^g^ Primary Health Care Services, Dubai Health Authority Dubai UAE; ^h^ Department of Family and Community Medicine, University of Gezira Gezira Sudan; ^i^ Department of Health System Development, WHO EMR Office Cairo Egypt; ^j^ WONCA East Mediterranean Region Amman Jordan; ^k^ Department of General Practice and Primary Health Care, University of Auckland Auckland New Zealand; ^l^ Department of Population Health and Primary Care, University of East Anglia Norwich UK; ^m^ Department of Community and Family Medicine, Fukushima Medical University Fukushima Japan

**Keywords:** Primary healthcare, community health services, family physicians, healthcare facilities, healthcare quality, access, health services

## Abstract

**Background:** Primary healthcare (PHC) is essential for equitable access and cost-effective healthcare. This makes PHC a key factor in the global strategy for universal health coverage (UHC). Implementing PHC requires an understanding of the health system under prevailing circumstances, but for most countries, no data are available.

**Objectives:** This paper describes and analyses the health systems of Bahrain, Egypt, Lebanon, Qatar, Sudan and the United Arab Emirates, in relation to PHC.

**Methods:** Data were collected during a workshop at the WONCA East Mediterranean Regional Conference in 2017. Academic family physicians (FP) presented their country, using the WONCA framework of 11 PowerPoint slides with queries of the country demographics, main health challenges, and the position of PHC in the health system.

**Results:** All six countries have improved the health of their populations, but currently face challenges of non-communicable diseases, aging populations and increasing costs. Main concerns were a lack of trained FPs in community settings, underuse of prevention and of equitable access to care. Countries differed in the extent to which this had resulted in coherent policy.

**Conclusion:** Priorities were (i) advocacy for community-based PHC to policymakers, including the importance of coordination of healthcare at the community level, and UHC to respond to the needs of populations; (ii) collaboration with universities to include PHC as a core component of every medical curriculum; (iii) collaboration with communities to improve public understanding of PHC; (iv) engagement with the private sector to focus on PHC and UHC.

Key Messages

- Primary healthcare (PHC) as a policy priority in eastern Mediterranean countries is hampered by policymakers’ lack of understanding the complex nature of PHC.
- Advocacy of PHC should stress its contribution to Universal Health Coverage.
- Professional training in the community setting is a priority to implement PHC.

## Introduction

Most countries experience significant challenges to their health systems, due to increasing health costs and diminished returns on healthcare investment for aging populations. Where primary healthcare (PHC) is formally structured in the health system, and professionals are educated in the primary care setting, the system realizes better population health at lower healthcare costs [1–4]. This has made PHC a global strategy to secure sustainable healthcare [5]. This policy strategy has been reinforced by the pursuit of universal health coverage (UHC) [6], as part of the UN sustainable development goals [7].

Implementing PHC policy asks for the application of general principles under prevailing local conditions [8], and builds PHC from the community level where it operates [9]. An understanding of the existing health system is essential in initiating reforms. This is available for Europe, Australia, New Zealand, and North America [4,8], but for many countries or regions data are scarce [9]. To address this, the World Organization of Family Doctors (WONCA) Working Party on Research took the initiative to document PHC around the world, and stimulate dialogues of how the values of PHC can be addressed within the constraints of different health systems [10]. Earlier studies documented the Asia-Pacific and South Asia regions and in Mexico [11–13]. From these studies, common challenges and priorities were identified to realise PHC and secure UHC—despite differences in culture, demography or history of health systems. This stresses their relevance for other regions, including Europe.

This paper is the first to document and critically appraise the health systems from the Eastern Mediterranean region, with the objective of identifying common strategies for strengthening PHC, and prioritizing regional collaboration, and exploring collaboration with the World Health Organization (WHO) Regional Office for the Eastern Mediterranean (EMR).

## Methods

A workshop at the 2017 WONCA East Mediterranean Regional Conference in Abu Dhabi compared the health systems of six WONCA member countries: Bahrain, Egypt, Lebanon, Qatar, Sudan and the United Arab Emirates (UAE) provided the data for this paper. Each selected an academic family physician (FP) to present their country, using the WONCA framework of 11 PowerPoint slides that focused on country demographics, the health system and the position of PHC, the country’s leading health challenges, strengths and weaknesses and lessons others could learn from their country [10]. They were free to concentrate on what, in their view, was the most important. All workshop presenters and moderators contributed to the discussion, directed to strategies to strengthen PHC and the contribution regional and international collaboration could make. Presentations (of which presenters provided a summary) and the discussions formed the basis of this article.

## Findings: country profiles

Bahrain has a population of 1.4 million served by 9.1 physicians per 10,000 inhabitants, working in the private and government sectors [14,15]. PHC was introduced in the 1950s and strengthened in 1983 through a four-year specialty-training programme for FPs in collaboration with the American University of Beirut, the Irish College of General Practitioners and The Royal College of Surgeons in Ireland. On graduation, each physician has to undergo the Arab Board Examination. This has enhanced the incorporation of the concept of family medicine and its acceptance by the public [16]. Over 24 PHC centres and three clinics have been established in the country geographically, with people registered according to their residential addresses. Health centres differ in the size of the population served, between less than 15,000 to 30,000–35,000. At present around 500 qualified FPs have graduated from the program, but nearly double this number are needed for the growing population. The main problem is the small capacity of the training program of 20 candidates annually.

Egypt has a population of 92.1 million with 28.3 physicians per 10,000 inhabitants [15,17]. PHC was established in the early 1940s based on general practice and maternity and child health services by the mid-1990s. There are approximately 5314 PHC facilities with 14,973 general practitioners and 256 certified FPs. Of these facilities, 61% implemented an FP approach based on formal accreditation [17]. Three types of facilities are in operation: family health units, family health centres, and district hospitals; with a PHC facility within less than 5 km for 95% of the population. This has resulted in nearly 91% children aged 18–29 months fully vaccinated [18]. The government has created a four-year FP fellowship training programme, while various universities did shape a postgraduate five-year training programme and some universities introduced PHC in their undergraduate curriculum. Despite the wide geographic distribution of PHC, there are still only 0.6 PHC centres and units, and less than 0.08 FPs per 10,000 population. The main challenges are the high out-of-pocket expenditure on health, low government spending and poor government vision on family practice, resulting in poor public health services that force most of the poorest to use private healthcare.

Lebanon is has a population of 5.8 million, with 31.9 physicians per 10,000 [15]. The health system is based on a public–private partnership with less than half the population covered by health insurance. The government covers the remaining for secondary and tertiary care, while PHC requires out of pocket payment. Private practice dominates, particularly in large cities [19]. There are five FP residency programmes but the number of practicing FPs is limited. There are approximately 950 dispensaries and PHC centres providing affordable healthcare to the poorer segments of the population. The government provides in-kind support to its 200 PHC centres, which are mainly managed by non-governmental organizations or municipalities [20]. Multidisciplinary teams provide comprehensive preventive and curative care (immunizations, child and reproductive care, oral health, provision of essential acute and chronic medications). Strategies taken to strengthen PHC are integrated management of mental health and non-communicable diseases; professional capacity building; accreditation of PHC centres; and introducing a health information system. This has resulted in improved quality and enhanced community engagement [21]. The first steps towards UHC were directed at individuals living below the poverty line. Serious challenges are the limited PHC budget, a further influx of refugees on top of the current one million from Syria [22], and the unstable economic and political situation.

Qatar has a population of 2.4 million, with 77.4 physicians per 10,000 inhabitants [15]. It launched a national health strategy in 2011, with PHC as its basis. This was followed-up by a *Vision* articulating the requirements of a healthy society to meet the country’s growth and development. In this, the Prince of Qatar decreed an independent funding and leading role for the PHC Corporation in 2012. The PHC Corporation is now responsible for 23 health centres, all accredited by Accreditation Canada International, in three different regions geographically distributed across the State of Qatar as a public provider. Its focus is on integrating high-quality curative services with promoting healthy living, and on community and patient engagement towards a healthier future. This is based on the family medicine model, through multidisciplinary teams that cover all age groups with a wide range of services (including prevention, management of chronic illness, rehabilitation support, and end of life care) in the community. There are currently 139 certified Qatari FPs, with 12 FPs graduating annually through the FP residency programme—which is insufficient to cover the country’s needs. This exemplifies some challenges Qatar is facing but at the same time captures a positive incentive towards PHC.

Sudan with a population of 39.6 million and 2.5 physicians per 10,000 started the PHC approach in 1976 to achieve rural extension for the then urban-based health services [15]. A significant issue is the shortage of health professionals and their background: there are six times more physicians than nurses, while most of the physicians are specialists (less than one third of generalists). On top of this comes the high turnover: a substantial number of FPs move to Saudi Arabia and the Gulf after graduation. An FP training programme was established in Sudan in 2008. To date, 375 FPs have graduated, of whom 174 still work in the country. In Gezira, there are 120 students enrolled in its FP master programme (with an additional 250 in public health) and 50–60 in Khartoum. Its programme pursues a family health approach led by three categories of PHC providers: FPs, medical assistants, and community health workers who operate basic health units and deliver essential PHC services. The health centre headed by FPs forms the first referral point for lower-level facilities. The community participates in the planning and performance of PHC. Currently, 109 FPs work in 84 health centres.

The UAE has an estimated population of 9.6 million of which Emirati nationals represent 19% [15]. Population growth and aging, together with an increase of medical tourism stretch the available facilities [23]. There are 19.3 physicians and 40.9 nurses and midwives per 10,000 inhabitants and well over 104 public and private hospitals and 1075 public and private outpatient clinics [15,24]. This includes 150 public centres and clinics, that provide PHC and in which 1004 physicians (363 residency trained FPs) practice with a further 40 PHC centres planned [25]. This has resulted in substantial progress in population health, in particular maternity and infant health, with all births attended by skilled health professionals. The FP residency training established in Dubai in 1993 is now extended to Al Ain, Abu-Dhabi, and Sharjah and the northern Emirates with four-year programmes recognized by the Arab Board of Medical Specialization. Collaboration with the UK Royal College of General Practitioners has improved the quality of these programmes. The annual output of the FP residency training programmes of 16 is insufficient to meet the demands of the UAE now and in the short-term, while the introduction of universal health insurance will further increase this demand.

## Discussion

Each country presented a unique situation, but all had PHC on their health agenda. From it, four themes emerged: (i) the gap between PHC facilities currently realized and what is required to serve their populations; (ii) problems amongst policymakers in understanding the complex nature of PHC and the importance of comprehensive policy to realize it; (iii) the importance of investing in the training of (future) professionals in the primary healthcare setting; and (iv) the importance of involving the private, next to the public sector in health reforms.

All six countries have made progress in the health of their populations but there were general concerns of an underuse of prevention and of equitable access to care. Countries differed in the realization of a coherent policy to address these issues. Bahrain and Sudan have developed a structure of community based PHC as the entry point of the health system, with a leading role for FPs, and Qatar has recently launched plans to strengthen community engagement in improving health outcomes. In Lebanon, the introduction of UHC is connected to the provision of community health services and directed at the poorest population. Other countries are aware of the need to introduce PHC in their health systems, but without a targeted policy to bring this about. This was exemplified in Egypt, with its long tradition of PHC that has improved maternity and child health [17]. Yet, as had also been seen in in other regions, the successful implementation of *limited PHC* (i.e. restricted to programs focussing on only a few health problems) has not evolved in a structure that integrates the broad field of prevention, treatment and support of all health problems in all individuals [12].

### Implications: integrated health policy

To achieve this integrated approach, a comprehensive health policy is required to regulate the health system, define the role and function of PHC, and make sure that professionals have the competence to fulfil this role. Of particular importance is here to include the private sector [25], as most healthcare (including that for the poorest segment of the population) is provided privately [26]. Public–private partnerships are common in the region, but not in health policy. Regulation and standardization of the private sector is required to stimulate collaboration and promotion of health, to address the needs of the populations. Related to this, PHC has to have the function to coordinate and horizontally integrate the provision of healthcare. Health policy should counter that every specialist can claim ‘primary health care’ and practice in the community.

### Implications: professional capacity building

For PHC to be able to lead the health system, training of professionals—including FPs—in the PHC setting is a mandatory precondition. Specialty training for FPs is provided by medical schools in the countries, but has a low priority, resulting in a shortage of FPs [27]. The strong focus on secondary and hospital-based care, with its high status amongst patients and public has been encountered in other regions as well [28]. Those who have completed specialty training experience their competences undervalued, which further stimulates a ‘brain drain’ to other countries and regions.

Another measure is to combine programmes for practitioners already working in the system to improve their skills with specialty training of residents, as recommended by the WHO EMRO [27].

### Implications: understanding PHC

For consistent policy, it is essential to understand the complex nature of PHC and of how it contributes to health systems. Unfortunately, policymakers consider PHC an ill-understood ‘black box’ [29], and that is where WHO EMRO has focussed its advocacy and where academic and professional PHC organizations can make a valuable contribution [27].

### Implications: towards regional collaboration

The shared challenges on the road to PHC implementation present a strong case for regional collaboration in which PHC leadership connects with policymakers and other stakeholders [25,30]. In this, a partnership with the powerful WHO EMRO office would offer an excellent opportunity for a regional action plan to realize UHC through PHC [26,27].

The priorities in PHC that emerged from this analysis concur with priorities in other regions—including Europe. Particularly relevant from a European perspective is how to align the private and public sectors in PHC development and how to secure a policy towards UHC that comprehensively addresses the health system regulations, professionals’ roles, and specialty training.

### Conclusion and key messages

Despite their differences in their socio-economic situation and track record of their health systems, the six countries face the same challenges in securing robust PHC and UHC. In conclusion, four priorities of joint action were identified:

- Advocacy for community-based PHC and UHC to policymakers, including the central role of PHC in coordinating healthcare at the community level. The experiences in Bahrain and Sudan can serve as models of success;
- Collaboration with university leaders and deans of medical schools to include PHC as a core component of every medical curriculum, followed by specialty training in PHC settings for those choosing a career as FP;
- Collaboration with patients and community leaders to inform the public about the role and function of PHC to improve its understanding. Collecting and displaying stories and experiences of patients can be of great value in this [13].
- As the private sector is a main outpatient health services provider, engaging the private health sector is inevitable to realize UHC.

## References

[1] Starfield B. Is primary care essential? Lancet. 1994;344:1129–1133. 7934497 10.1016/s0140-6736(94)90634-3

[2] Starfield B, Shi L, Macinko J. Contribution of primary care to health systems and health. Milbank Q. 2005;83:457–502. 16202000 10.1111/j.1468-0009.2005.00409.x PMC2690145

[3] Hansen J, Groenewegen PP, Boerma GW, et al Living in a country with a strong primary care system is beneficial to people with chronic conditions. Health Aff (Millwood). 2015;34:1531–1537. 26355055 10.1377/hlthaff.2015.0582

[4] Kringos D, The strength of primary care in Europe [thesis]. Utrecht, University of Utrecht, 2012 [Internet]; [cited 2017 Feb 28]. Available from: http://www.nivel.nl/sites/default/files/bestanden/Proefschrift-Dionne-Kringos-The-strength-of-primary-care.pdf

[5] World Health Organization The World Health Report 2008: primary health care, now more than ever. Geneva: WHO; 2008 [Internet]; [cited 2017 Feb 28]. Available from: http://www.who.int/whr/2008/en/

[6] United Nations Sustainable Development Goals. 2017 [Internet]; [cited 2017 Apr 19]. Available from: http://www.un.org/sustainabledevelopment/sustainable-development-goals/

[7] WHO Universal Health Coverage. 2017 [Internet]; [cited 2017 Feb 13]. Available from: http://www.who.int/universal_health_coverage/en/

[8] Journal of the American Board of Family Medicine. 2012;25(Suppl 1). [Internet]; [cited 2017 Mar 4] Available from: http://www.jabfm.org/content/25/Suppl_1.toc

[9] van Weel C, Turnbull D, Whitehead E, et al International collaboration in innovating health systems. Ann Fam Med. 2015;13:86–87. 25583898 10.1370/afm.1751 PMC4291271

[10] WONCA research working party multi-national plenary panel project. 2017. [Internet]; [cited 2017 March 04]. Available from: http://www.globalfamilydoctor.com/groups/WorkingParties/Research/plenarypanelprojectresourcedocuments.aspx

[11] van Weel C, Kassai R, Tsoi GWW, et al Evolving health policy for primary care in the Asia Pacific region. Br J Gen Pract. 2016;66:e451–e453. 27231305 10.3399/bjgp16X685513 PMC4871311

[12] van Weel C, Kassai R, Qidwai W, et al Primary healthcare policy implementation in South Asia. BMJ Glob Health. 2016;1:e000057. 10.1136/bmjgh-2016-000057 PMC5321321 28588938

[13] van Weel C, Turnbull D, Ramirez J, et al Supporting health reforms in Mexico: experiences and suggestions from an international primary health care conference. Ann Fam Med. 2016;14:280–281.27185004

[14] Alnasir F. Ageing and pattern of population changes in the developing countries. MEJAA. 2015;12:26–32.

[15] WHO EMRO. Country health profiles 2017 [Internet]; [cited 2017 Sep 6]. Available from: http://www.emro.who.int/entity/statistics/statistics.html

[16] Alnasir F. Family medicine in the Arab world? Is it a luxury? J Bahrain Med Soc. 2009;21:191–192.

[17] Government of Egypt CAPMAS statistical yearbook (after MOHP). 2016 [Internet]; [cited 2017 Mar 23]. Available from: http://www.capmas.gov.eg/Pages/StaticPages.aspx?page_id=5034

[18] El-Zanaty F, et al. Egypt Demographic and Health Survey 2014 Cairo and Maryland: Ministry of Health and Population, the DHS Program, ICF International; 2014 [Internet]; Available from: http://dhsprogram.com/pubs/pdf/PR54/PR54.pdf

[19] World Health Organization Country cooperation strategy for WHO and Lebanon 2010–2015. 2017 [Internet]; [cited 2017 Mar 23]. Available from: http://www.who.int/country-cooperation/publications/en/

[20] Ammar W, Kdouh O, Hammoud R, et al Health system reliance: Lebanon and the Syrian refugee crisis. J Glob Health. 2016;6:020704. 28154758 10.7189/jogh.06.020704 PMC5234495

[21] El-Jardali F, Hemadeh R, Jaafar M, et al The impact of accreditation of primary health care centres: successes, challenges and policy implications as perceived by primary health care providers and directors in Lebanon. BMC Health Serv Res. 2014;14:86. 24568632 10.1186/1472-6963-14-86 PMC3946059

[22] United Nations High Commissioner on Refugees Syria Regional Refugee Response. Inter-agency information sharing portal. Syria: UNHCR 2017 [Internet]; [cited 2017 Mar 23]. Available from: http://data.unhcr.org/syrianrefugees/country.php?id=122

[23] Gulf News. March 30, 2017 Dubai prepares for influx of 1.3 m medical tourists by 2021. 2017 [Internet]; [cited 2017 Mar 30]. Available from: http://gulfnews.com/news/uae/health/dubai-prepares-for-influx-of-1-3m-medical-tourists-by-2021-1.1818558

[24] The U.S.–U.A.E. Business Council The U.A.E. healthcare sector: an update. September 2016 [Internet]; [cited 2017 Mar 30]. Available from: http://usuaebusiness.org/wp-content/uploads/2016/09/Healthcare-Report-Final.pdf

[25] UAE Interact 2017 [Internet]; [cited 2017 Mar 30]. Available from: http://www.uaeinteract.com/society/health.asp

[26] World Health Organization Regional Office for the Eastern Mediterranean. Analysis of the private health sector in countries of the Eastern Mediterranean. 2014 [Internet]; [cited 2014 Apr 03]. Available from: http://applications.emro.who.int/dsaf/EMROPUB_2014_EN_1790.pdf?ua=1

[27] Scaling up family practice: progressing towards universal health coverage, EM/RC63/Tech.Disc.1. 2016. [Internet]; [cited 2017 Sept 04]. Available from: http://www.emro.who.int/about-who/rc63/documentation.html

[28] van Weel C, Kassai R. Expanding primary care in South and East Asia. BMJ. 2017;356:j634. 28242604 10.1136/bmj.j634

[29] Primary Health Care Performance Initiative. 2017 Measuring PHC. [Internet]; [cited 2017 Mar 8]. Available from: http://phcperformanceinitiative.org/about-us/measuring-phc

[30] Rawaf S, Qidwai W, Khoja TAM, et al. New leadership model for family physicians in the Eastern Mediterranean region: a pilot study across selected countries. J Fam Med. 2017;4:1107.

